# Identification Performance During Quarantine by COVID-19 Pandemic: Influence of Emotional Variables and Sleep Quality

**DOI:** 10.3389/fpsyg.2021.691583

**Published:** 2021-10-15

**Authors:** Facundo A. Urreta Benítez, Candela S. Leon, Matías Bonilla, Pablo Ezequiel Flores-Kanter, Cecilia Forcato

**Affiliations:** ^1^Laboratorio de Sueño y Memoria, Departamento de Ciencias de la Vida, Instituto Tecnológico de Buenos Aires (ITBA), Buenos Aires, Argentina; ^2^Consejo Nacional de Investigaciones Científicas y Tecnológicas (CONICET), Buenos Aires, Argentina; ^3^Innocence Project Argentina, Buenos Aires, Argentina

**Keywords:** recognition, lineup, episodic memory, anxiety, depression, sleep, COVID-19

## Abstract

The COVID-19 pandemic has caused major disruptions in people’s lives around the globe. Sleep habits and emotional balance have been disturbed in a way that could be comparable to the havoc caused by a deep personal crisis or a traumatic experience. This unfortunate situation provides a unique context in which to study the impact of these imbalances on cognitive processes. In particular, the field of eyewitness science could benefit from these conditions, since they are also often present in crime victims, but can only be generated in the laboratory up to a certain ethical and practical limit. For several decades, eyewitness studies have tried to discover what variables affect people’s ability to properly recognize faces. However, the disparity of experimental designs and the limitations of laboratory work could be contributing to the lack of consensus around several factors, such as sleep, anxiety, and depression. Therefore, the possibility of observing the influence of these agents in natural contexts could shed light on this discussion. Here, we perform simple and repeated lineups with witnesses of mock-crime, considering the conditions related to the COVID-19 pandemic, which to some extent allow emulating the deterioration in general well-being that often afflicts crime victims. For this, 72 participants completed symptomatology scales, and watched a video portraying a staged violent episode. Subsequently, they gave testimony and participated in two lineups, in which we manipulated the presence/absence of the perpetrator, to recreate critical scenarios for the appearance of false recognitions. We found an increase in recognition errors in those individuals who did not have access to the perpetrator during the Initial lineup. Additionally, the conditions of the pandemic appear to have adversely affected the ability to witness and accurately perform lineups. These results reaffirm the need to move toward the standardization of research practices and methods for assessing testimonial evidence, especially in relation to the results of the lineups. Considering the degree of fallibility of these processes can lead to a reduction of wrongful convictions.

## Introduction

Since the beginning of the COVID-19 pandemic, mental health surveys have been conducted consistently around the world. The concern arises from the fact that social isolation, confinement, and sedentary lifestyle are directly associated with a broad range of mental disorders (Shah et al., 2021). The results of these studies indicate a significant prevalence of negative feelings, derived from the fear of being infected, economic instability, frustration, and boredom (among others) (Brooks et al., 2020). As a result of this unfavorable situation, intense symptoms of anxiety (a psychophysiologic sign of stress, Robinson, 1990) and depression have been observed in large segments of the population (Ozamiz-Etxebarria et al., 2020; Shah et al., 2021; Sheridan et al., 2021), as well as significant disturbances in sleep habits (Barros et al., 2020; Jahrami et al., 2021). This unfortunate situation provides a unique opportunity to study how these disturbances affect various human activities, especially in those areas where the lack of consensus could be a direct consequence of ethical and methodological limitations. Such is the case of eyewitness studies.

A difficulty that constantly appears throughout most of the background in eyewitness science is the limitation (fundamentally ethical) to produce ecological conditions in the laboratory (Valentine and Mesout, 2009), that is, levels of anxiety, depression, or sleep loss like those that could be observed in crime victims. The need to find a way to compensate for these methodological flaws becomes evident when contemplating the consequences of the scarcity of applicable knowledge.

According to information from the Innocence Project organization^1^, 72% of wrongful convictions in the United States are strongly determined by incorrect identifications in lineups. Even acting in good faith, people may misrecognize an individual in a lineup, due to various environmental and personal factors. In order to understand this, we must understand memory as highly malleable and subject to distortion and not as a video camera that faithfully reproduces the past (Clifasefi et al., 2007). These distortions can lead to the formation of false memories, that is, memories of events that never occurred, or memories with added or altered details (Loftus, 2003; Newman and Lindsay, 2009).

The formation of false memories constantly occurs in everyday life. Without realizing it, people incorporate details and characters that come from dreams, external suggestions, or confusion to their anecdotes (Reyna et al., 2016). In terms of the ability of making decisions in lineups and describing events, memory errors can have drastic consequences (mainly in the legal field). Therefore, it is imperative to discover what processes favor their appearance and how they can be reduced.

The emotional conditions of individuals are some of the most studied factors for their influence on the ability to recognize faces (Edelstein et al., 2004). In particular, the impact of a witness’s level of stress has been extensively addressed, considering the high stress that criminal acts, and subsequent police processes can generate (Dobson and Markham, 1992; Deffenbacher et al., 2004).

The models that relate stress and general cognitive performance have become more complex over the years, to capture more and more nuances, and account for the vastly disparate results in experimentation. This complexity is transferred directly to eyewitness studies, in general, the results found in the existing literature are mixed (Valentine and Mesout, 2009). Evidence of a facilitating effect has been found, in which subjects under higher stress have more accurate memories of a witnessed event (Lindberg et al., 2001). Yuille et al. (1994) presented a group of police recruits with a simulation task in which a situation of greater or lesser stress was set. In the following weeks, the participants were interviewed, and it was observed that those who had participated in the high-stress set-up, reported more precise details. Opposite findings have also been made (Pezdek et al., 2020), in which witness performance worsens as stress arises. Morgan et al. (2004) studied the performance in lineups of a group of soldiers enrolled in a military survival academy. After a 12-h confinement in a simulated prison camp, the participants experienced a low- or high-stress interrogation (the interrogators were more or less aggressive). A day later, the soldiers went through a lineup trying to identify their interrogators. A dramatic decrease in accuracy was observed in the high-stress group, compared to the low-stress group. According to Tyng et al. (2017) stress can affect performance on memory tasks, both positively and negatively. This depends mainly on the intensity of the stimulus, type of memory involved, and the phase of the memory process where the excitation is applied (Deffenbacher et al., 2004). A very popular current theoretical model proposes that the release of hormones during stress (particularly catecholamines and glucocorticoids) turns the stressed organism in a “memory formation mode” that prioritizes the encoding and storage, to the detriment of retrieval. This mechanism has an adaptive value, since it prioritizes the acquisition of information in a potentially dangerous environment and can explain the performance of our participants, who learned better, but retrieved worse at higher levels of arousal (Schwabe et al., 2012).

Depressive mood is another emotional disturbance that usually appears in witnesses and crime victims (Norris and Kaniasty, 1994). This affliction can persist for long periods (it can encompass the entire judicial process) and can result into a major depressive disorder, interpersonal problems, and even lead to suicide (Rounding et al., 2014). There is evidence suggesting that chronically negative mood states increase the possibility of selecting the target in a lineup. Rounding et al. (2014) showed a group of healthy subjects a series of images of faces, while assessing the possible presence and intensity of their depressive symptoms. A week later, the same individuals had to make an identification on a series of six-person lineups, attempting to recognize the faces previously observed. The results showed that those with mild sustained dysphoria generally had greater accuracy than those without symptoms and those severely depressed. This effect is usually stable and is not affected by acute mood changes, whether it is positive feelings or new depressants (unless the latter are very intense). In general, it is considered that individuals with high depressive symptoms elaborate the information stored in memory in an active and biased way, that is, they tend to select the details that make up negative events with violent or unpleasant elements, which in a certain way reaffirm their biased perception of reality (Watkins et al., 1996). As a result, one of the most robust findings in the literature on depression is that depressed subjects had a stronger and more persistent memory of negative events, while they more easily forget the positive or pleasant (Gotlib and Joormann, 2010).

Outside emotional factors, there are variables related to the individual that must be considered when studying their performance in lineups. Among them, sleep is one of fundamental importance, given its role in the acquisition, consolidation, and integration of new information (Rasch and Born, 2013). In general aspects, it is quite clear that sleep is beneficial for memory (Walker, 2009). However, when observing this relationship in greater depth, it becomes evident not only that this facilitating effect differentially affects the different memory phases, but that it is also capable of producing undesired results. Sleep favors subsequent memory acquisition while sleep disturbances can lead to an encoding decline (Van Der Werf et al., 2009) and this effect is usually explained by the synaptic homeostasis hypothesis (Tononi and Cirelli, 2003). According to this hypothesis, sleep favors the decay of weak synaptic connections formed during wakefulness, performing a downscaling of the synapses, which benefits strong connections (increasing the signal-noise ratio), and highlighting the information that is most valuable. Because of this downscaling, adequate sleep translates into increased encoding ability during later wakefulness. However, the beneficial effect of sleep does not stop there. Sleep also improves memory consolidation (Born and Wilhelm, 2012) through active consolidation processes. This theory proposes that during sleep, specifically during Slow Wave Sleep (SWS) recently acquired memories are spontaneously reactivated, promoting the gradual redistribution of hippocampus dependent memories from the hippocampus to neocortical areas where they will be stored in long term networks. Furthermore, Rapid Eye Movement (REM) sleep favors integration of memories (Payne, 2014). An integral part of this process is the joint reactivation of new and old memories, to find overlaps and extract central ideas that link the information units to each other (Diekelmann and Born, 2010). These central ideas tend to be more durable in time than specific details (these tend to disappear more easily) and have been identified as possible causes of false memories (Payne et al., 2009). Thus, when we remember a certain event, it is much easier to access its meaning than its details. For this reason, when producing a detailed account, we may find blank spaces, that is, elements that are missing. Furthermore, the stronger a central idea (gist) is, and the weaker the recall of details, the more we will tend to produce false memories (Brainerd and Reyna, 2002).

These claims about the impact of sleep on general memory processes have considerable consensus and are well documented. However, when attempting to translate these results into the specific field of eyewitness science, drawbacks arise. Studies are scarce, and the results are mixed. For example, Stepan et al. (2017) showed the participants a mock-criminal video and performed a photographic lineup 12 h after in the presence or absence of the target. The participants that slept between the training and the testing sessions had a better performance rejecting the innocent when the perpetrator was absent in the lineup. Nevertheless, in a similar procedure, Morgan et al. (2019) did not observe differences between groups of participants that either slept after the training or remained awake.

When considering the ability to recall details, it has been observed that the quality of sleep prior to the mock-crime influences performance, in fact, as the quality of sleep decreases, the ability to recall details also decreases. Thorley (2013) showed a video of a simulated crime to a group of subjects who reported their level of sleepiness. They also completed sleep quality scales, referring to the night prior to the experiment. In a later recall of what was watched in the video, it was observed that as sleep quality decreases and sleepiness increases, individuals tend to report less detail. This result goes in line with several studies showing that sleep deprivation impairs memory acquisition during subsequent wakefulness (Kaida et al., 2015). Furthermore, the relationship between confidence in choice and accuracy decreases under conditions of sleep deprivation (Blagrove and Akehurst, 2000).

As has been observed there are few clear trends and consensus still needs to be reached on multiple issues. For this reason, locating the experiment in a natural environment, which spontaneously presents one or more of these characteristics (Morgan et al., 2013) could be beneficial, and bring some clarity about misidentifications.

A particular problem very present in police practices in some regions of the world is that of repeated lineups. Particularly in the case of Latin America, these procedures are applied despite being strongly contraindicated. Repeated lineups have been extensively studied and are generally considered unreliable. During the second lineup, people are often retroactively influenced by the first (Steblay and Dysart, 2016). This may be due to the occurrence of a “compromise effect,” that is, a person tends to repeat their choices, to show consistency to themselves and others (Valentine et al., 2012; Lin et al., 2019). It can also be due to the “transference effect” (Loftus, 1976), the inability of an eyewitness to distinguish between a familiar but innocent person, from the actual criminal that was observed at the scene of crime (Ross et al., 1994). Finally, it can also be caused by the process of memory reconsolidation (especially in the absence of the perpetrator during the first lineup). That is, during the initial lineup some faces of the foils could have similar features as the target, triggering a prediction error, i.e., the mismatch between what is predicted according to previous experiences and what is encountered during re-exposition allowing memory labilization (Forcato et al., 2020). In this case, the memory would be updated during reconsolidation, incorporating erroneous information from the faces present in the lineup into the original memory.

However, studies on multiple identifications often do not consider the impact of sleep and emotional variables on the process (since observation tends to focus on repetition itself). Given the influence that these variables seem to have on simple identifications, and the fact that repeated lineups are still practiced in some countries, it is of interest to contemplate this case from an exploratory perspective.

In this exploratory study, we will analyze the impact on performance in simple and repeated lineups, of emotional states and sleep habits during the lockdown related to the COVID-19 pandemic.

Finally, the influence of the same variables in tasks of free recall and chronological order of images will be studied, seeking to determine if the relationships are repeated through different memory modalities.

To this end, two groups of subjects completed psychometric scales, watched a video of an incident at a conference, and gave their oral testimony (day 1). 24 h later, they tried to identify the perpetrator in a photographic lineup, in the presence (With perpetrator group) or absence (Without perpetrator group) of the perpetrator (day 2). On day 8 both groups carried out a definitive lineup in present condition. Additionally, they gave a final testimony, and were tested for the episodic temporal order of the event.

## Materials and Methods

The participants were 78 Argentines recruited online through the official social networks of the Sleep and Memory Lab. Applicants underwent a prior online interview with the experimenter, to ensure that they met the inclusion criteria. They also had to demonstrate that they had the appropriate technical resources (PC and fast enough Internet etc.), and basic knowledge of how to use them. The sample size was decided according to previous studies sharing similar designs (Wells, 1984; Steblay et al., 2013). These studies with comparable sample size have demonstrated significant effects of behavioral intervention, suggesting the reproducibility of these effects on memory with similar sample size.

For the collection of sociodemographic data and symptomatological scales, the Google Forms platform was used. This form of data collection has been shown to be equivalent to traditional forms of collection (Weigold et al., 2013). Then, the experiment was carried out through the Google Meet video calling platform with the experimenter guiding the entire process. It was controlled that all people have access to a computer screen (not cell phone) and that they have quality internet connection. The experiments were approved by the Alberto C. Taquini Biomedical Research Ethics Committee. 6 participants were excluded from the data analyzes because they only completed the first session of the experiment (day 1) and did not show up to the following meetings. The final sample consisted of 72 subjects (Age *M* = 29 ± 6, years, Table 1). Inclusion criteria: The participants stated that they were not ill during the experiment, did not suffer from mental disorders, took psychiatric medication, or had sleep disorders. The experiments were carried out between the months of April and September 2020, within the period of preventive and mandatory social isolation in Argentina.

**TABLE 1:** Sociodemographic data.

		With perpetrator group	Without perpetrator group
N		39	33
Age		29.33 ± 5.61	28.45 ± 7.16
Gender	Females	79.48%	78.78%
	Males	17.94%	21.21%
	Non-binary	2.56%	
Education	High school graduates	10.25%	9.09%
	College students	17.94%	33.33%
	College graduates	71.79%	51.51%

*Number of participants in each group, mean age ± SD, percentage of different genders, and education level.*

### Procedure

The entire study was conducted online, at the beginning of each session, the participants entered a video call with the experimenter, who provided instructions, showed the stimuli through streaming, and supervised the tasks.

**FIGURE 1:**
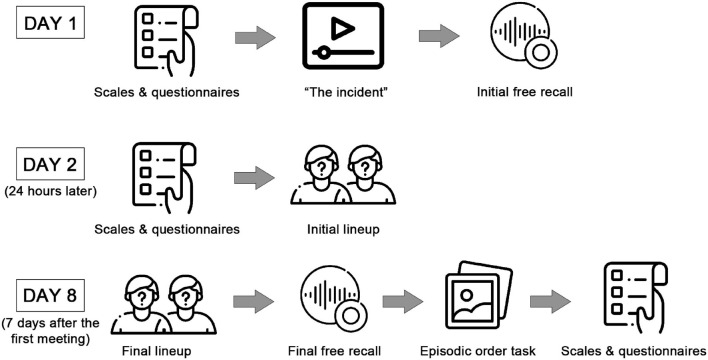
Experimental procedures. The scales and questionnaires included the sociodemographic questionnaire, State-Trait Anxiety Inventory, Beck Depression Inventory (BDI-II), and the Pittsburgh Sleep Quality Index (PSQI). The incident stands for the video of a perpetrator entering a conference. The initial lineup was formed by the presentation of 6 photos where the perpetrator could be present (With perpetrator group) or absent (Without perpetrator group). During the final lineup 6 photos were shown, including the perpetrator’s. Icons “To do list,” “Video player,” “Recording,” “Suspect,” and “picture” made by Freepik [https://www.flaticon.com/authors/freepik] from www.flaticon.com.

On day 1, after signing the informed consent, they completed the first part of the sociodemographic questionnaire, State-Trait Anxiety Inventory (STAI-Y), and Beck Depression Inventory (BDI-II). Immediately after, they watched a video called “The Incident,” featuring an individual acting aggressively in front of a crowd, and after that, the Initial free recall was performed. 24 h later (day 2), the participants completed the Stanford Sleepiness Scale (SSS), the State Anxiety Inventory, and carried out the Initial lineup. On day 8, the participants performed the Final lineup. Immediately after, a Final free recall of the video watched on day 1 was executed. Finally, they completed the Chronological order task, the Pittsburgh Sleep Quality Index (PSQI), the STAI-Y, and the second part of the sociodemographic questionnaire (Figure 1).

### The Incident (Video)

The video showed a conference in a room with numerous people. After about 30 s, a young man broke into the meeting to deliver a message. At that moment he argued with the main speaker of the talk, and became violent, yelling, and throwing objects to the ground. After this, he withdraws muttering. The speaker tried to retake the talk, and the video ended. It was filmed in high definition, by three cameras that alternated presenting a general panorama of the front of the room. It took 90 s.

### Initial Lineup

Subjects were presented with an image, simultaneously showing the photographs of 6 bearded males of similar ages and builds, randomly ordered, in black and white, numbered from left to right from one to six, in the form of a typical six-person lineup (lineup consisted of 5 foils and the perpetrator). For the Without perpetrator group, the perpetrator was extracted and another man with similar characteristics was added (lineup consisted of 6 foils). The participants were asked to observe the image for as long as necessary, noting the number that accompanies each photo. It was instructed that they had to identify the person who had broken into the talk or reject the lineup. The subjects received an unbiased instruction, which indicates the possibility of the absence of the perpetrator (“Now you are going to see a lineup with six photos, among which the person who broke into the video you saw yesterday may or may not be found. Take your time to see them. If you identify the suspect, I will ask you to tell me the number that accompanies his photo. If you consider that he is not present, tell me”). In response, they provided a number, or rejected the lineup. Immediately afterward, they were asked for an estimate of the degree of confidence in their own decision, with a number between zero and one hundred, representing with zero the absolute lack of confidence.

A total of 40 participants were recruited to assess an online fairness test of the with perpetrator six-persons lineup (lineup consisted of 5 foils and the perpetrator), and 64 participants for the six-person lineup that had no perpetrator, via a mock witness paradigm (lineup consisted of 6 foils) (Malpass and Lindsay, 1999). A group of simulated witnesses, who have not witnessed the crime video and who did not know the identity of the perpetrator, received a brief description of the perpetrator, and were asked to select the suspect from the list based on this description. For the lineup to be considered fair, the mock witnesses should not be able to identify the suspect at a rate greater than chance (lineup bias), and the distribution of their choices should be spread equally over the lineup members (lineup size, Brigham et al., 1999). In order to measure the lineup size, the Acceptable Lineup Members technique (ALM) was used (Malpass and Devine, 1983). A total of 75% was the minimum percentage of the probability expectation considered acceptable (Brigham et al., 1990). The Functional Size was used to measure the lineup bias (Wells et al., 1979).

In the lineup that included the perpetrator, an ALM of 3.40 and a Functional Size of 5 were obtained. In the lineup without the perpetrator an ALM of 2.50 was obtained.

### Initial and Final Free Recall

The participants were instructed to describe in as much detail as possible what they had watched in the video, mentioning that it might include dialogues, characteristics of the people (clothes and physical qualities, etc.) and the place, among other elements. The free recall was recorded, and the total number of details was counted and classified according to their veracity in correct and incorrect details. When counting the number of details, we considered actions, persons, objects, and elements of the environment. Every detail was counted only once, no matter how many times it was repeated in history. The instruction was “Now I’m going to ask you to describe, in as much detail as possible, what you have watched in the video. You can include dialogues, characteristics of the people (clothes and physical characteristics, etc.) and the place. I am going to record everything you say with the recorder.

### Final Lineup

During this test, the lineups of both groups included the perpetrator. It was similar to the Initial lineup, but the set of photographs was personalized, so that each subject could repeat their previous choice. Within the group of 6 photos, there were 3 new faces, and 3 previously seen (among which was the suspect chosen in the Initial lineup, if one has been chosen). On this occasion, both groups had access to the perpetrator and 5 foils, and the order of the photos was semi-randomized, so that none of the previously seen individuals occupied the same place. The fairness control of this lineup was carried out in the same way as in the Initial lineup and 40 mock witnesses were used. It obtained an ALM of 4.13 and a Functional Size of 5.

### Socio-Demographic Questionnaire

It included contact information, sex, age, educational level, occupation, cohabitation group, intake of medication, and presence of sleep disorders.

### State-Trait Anxiety Inventory

Based on a 4-point Likert scale, it contains 40 questions that are used to estimate two types of anxiety: state anxiety (the level of anxiety experienced at the time of performing the task) and trait anxiety (the personality-integrated anxiety of the individual) (Spielberger et al., 1970). The adaptation of this test for Argentina was used (Leibovich de Figueroa, 1991).

### Beck Depression Inventory

It is a multiple-choice inventory used to measure severity of depressive symptoms during the past 2 weeks. It is made up of 21 items that cover emotional, cognitive, and behavioral aspects (BDI-II; Beck et al., 1996).

### Chronological Order Task

The participants were provided with 5 images semi-randomly extracted from the video, and they were asked to order them chronologically, starting with the one that was observed first. The five images were presented simultaneously and the time to respond was unlimited. The task was scored considering the performance in terms of two factors: absolute location (that the first observed image was assigned to place 1) and relative location (that the first observed image was assigned to a position prior to the second observed image). The result obtained varies between 0 and 5, a higher score represents a better performance.

### Pittsburgh Sleep Quality Index

It is a self-administered questionnaire that assesses the quality of sleep integrating several factors, such as its latency, duration, and efficiency. Each component receives a score between 0 and 3, and is subsequently added to the others, having a result of between 0 and 21 points. Higher scores represent poorer quality of sleep (Buysse et al., 1989).

### Statistical Analysis

Statistical analyzes were carried out in the statistical software IBM SPSS Statistics 25, and RStudio Version 1.3.1073. The scores of the three symptomatology scales (STAI-Y, BDI-II, PSQI) were transformed into categories above and below average (low/high) in a similar way to the procedure of Valentine and Mesout (2009). The results of the Initial lineup for the With perpetrator group were considered: “target selection” if they selected the suspect, “foil selection” if they selected a wrong suspect and “incorrect rejection” if they rejected the lineup. In the case of the Without perpetrator group, the results of the Initial lineup were considered as “correct rejection” if they rejected the lineup and “foil selection” if they selected a wrong suspect. The results of the Final lineup were considered in the same way for both groups: “target selection” if they selected the suspect, “incorrect rejection” if they rejected the lineup and “foil selection” if they selected a foil. Considering the low number of participants who did not select anyone, “foil selection” and “incorrect rejection” were analyzed together. The score obtained from the Chronological order task was used as a direct value. The correct, incorrect, and total details were used as direct values. Additionally, the difference in the number of details (total, correct and incorrect) between day 1 and day 8 was treated as a direct value and as a proportion (memory change). We referred to the set of variables related to recall as: recall variables.

The frequency of target selection for the With/Without perpetrator groups, in the Initial and Final lineup, was analyzed with Pearson’s chi-squared test. We further calculated the Odds ratio. Additionally, chi-squared was used to compare the frequency of target selections of the subgroups (within With/Without perpetrator) of high or low anxiety, depression and quality of sleep, both in the Initial lineup and in the Final lineup, between them and against the chance level.

The recall variables were analyzed with two-tailed T-test comparing the high and low level of the symptomatology variables. The score of the chronological order task was related to symptomatology variables in the same way.

A paired *t*-test was used to compare recall variables between Initial and Final free recall. For the non-parametric variables, we used the Wilcoxon Signed-Ranks Test.

All tests were performed with a fixed alpha of 5%.

Additionally, to evaluate the relationship between the accuracy of the elections and the confidence attributed to them, two types of analysis were carried out. Initially, Point Biserial Correlation was applied (Krug, 2007) and then CAC curves were performed. To calculate the value of the correct proportion corresponding to each confidence level (low 0–50%, medium 60–80%, or high 90–100%) the following formula was used: # Correct identifications/# Correct identifications + # Incorrect identifications (Mickes, 2015).

## Results

### Lineup Recognition

**FIGURE 2:**
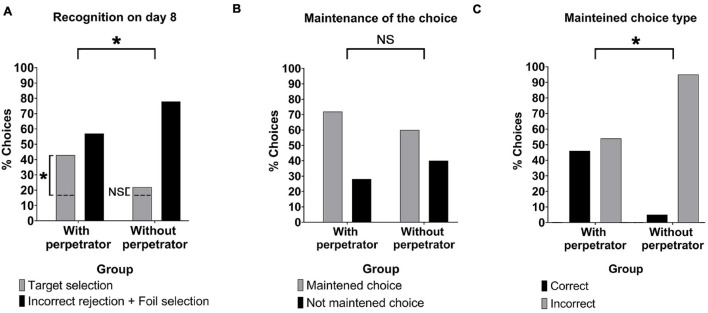
Lineup recognition. **(A)** Percentage of target selection and incorrect rejection + foil selection for the With perpetrator and Without perpetrator groups on day 8. With perpetrator group: target selection (*N* = 17), incorrect rejection (*N* = 0), and foil selection (*N* = 22). Without perpetrator group: target selection (*N* = 7), incorrect rejection (*N* = 1), and foil selection (*N* = 25). **(B)** Percentage of maintained and not maintained choices between day 2 and 8 for the With perpetrator and Without perpetrator groups. With perpetrator group: maintained choices (*N* = 28), not maintained choices (*N* = 11). Without perpetrator group: maintained choices (*N* = 20) and not maintained choices (*N* = 13). **(C)** Percentage of correct and incorrect maintained choices. With perpetrator group: correct (target selection/target selection, *N* = 12); incorrect (incorrect rejection/foil selection, *N* = 4 and foil selection/foil selection, *N* = 17). Without perpetrator group: correct (correct rejection/target selection, *N* = 1); incorrect (foil selection/foil selection, *N* = 24). The following choices were not present in the sample: incorrect rejection/incorrect rejection (for the With perpetrator group) and correct rejection/target selection and foil selection/incorrect rejection (for Without perpetrator group). *p < 0.05; NS, *p* > 0.05. The dashed lines stand for the chance level (17% of correct responses).

Regarding the repetition of the lineup, we observed that in the With perpetrator group 43% (*N* = 17) of the participants selected the target on Day 8, significantly higher than the 22% (*N* = 7) selected by the Without perpetrator group [Figure 2A, χ*2*(1) = 4.03, *p* = 0.045, φc = 0.24]. Thus, the target selection was alarmingly low for both groups. However, if we consider that by chance 17% (one sixth) of the subjects would select the target, we observed that the With perpetrator group was significantly higher than the chance level [χ*2*(2) = 6.02, *p* = 0.01, φc = 0.28]. This difference was not observed for the Without perpetrator group [χ2(2) = 0.10, *p* = 0.76, φc = 0.04]. Furthermore, based on the odds ratio, the odds that a subject would recognize the target on Day 8 would be 2.96 times higher if the perpetrator had been available during recognition on Day 2.

There were no significant differences between groups for the maintained choices between the recognition at Day 2 and the test at Day 8 (choose the same photo in both lineups) (Figure 2B, With perpetrator group: 72% (28), Without perpetrator group: 60% (20) [χ*2*(2) = 1.01, *p* = 0.32]. However, within these repeated identifications, the rate of correct choices was different for both groups. While the With perpetrator group showed that 46% (*N* = 13) of their repeated choices were correct (target selection-target selection), the Without perpetrator group showed that only one of their maintained choices was correct (correct rejection-target selection, Figure 2C, χ*2*(2) = 9.69, *p* < 0.01). It is important to consider that in the case of the Without perpetrator group a maintained correct identification would imply that the participant would have rejected the foils on Day 2 and have chosen the perpetrator on Day 8 while participants in the With perpetrator group have access to the perpetrator in both tests. We further analyzed the incorrectly maintained choices of the Without perpetrator group. We observed that 39% (*N* = 12) of the participants did not repeat the same choice in the Final lineup. However, 61% (*N* = 19) chose the same foil twice, significantly different to the choice level [χ2(1) = 13.33, *p* = 0.0003, φc = 0.46].

Regarding the Initial lineup, 36% (14) of the participants in the With perpetrator group achieved a target selection in the Initial lineup, while only 6% (*N* = 2) of the Without perpetrator group correctly rejected the lineup.

**FIGURE 3:**
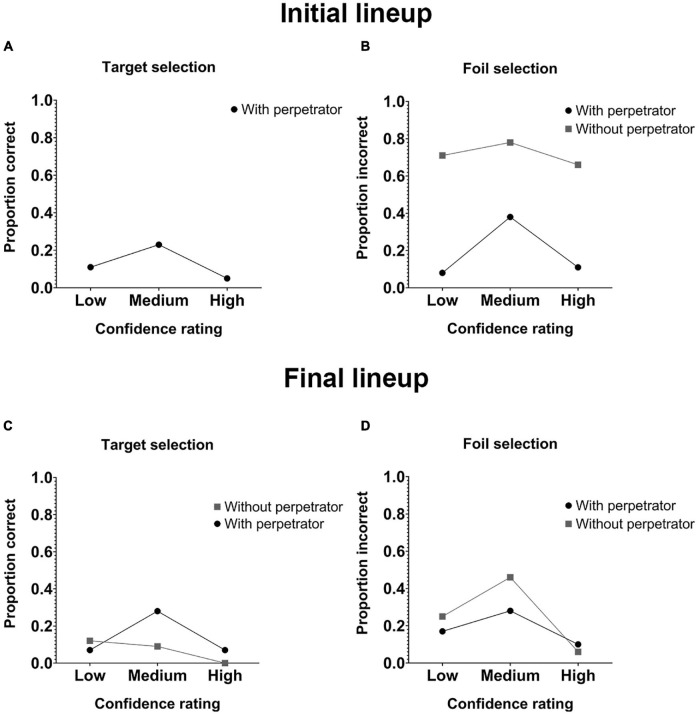
CAC curves for participants who made an election (choosers) in the Initial and Final lineups. **(A)** CAC curves for the target selection of the With perpetrator group of the Initial lineup. **(B)** CAC curves for the foil selection of the With perpetrator and Without perpetrator groups of the Initial lineup. **(C)** CAC curves for the target selection of the With perpetrator and Without perpetrator groups of the Final lineup. **(D)** CAC curves for the foil selection of the With perpetrator and Without perpetrator groups of the Final lineup. The perpetrator was included in the Final Lineup of both groups.

To verify the accuracy-confidence relationship in the Initial and Final lineup, Point Biserial Correlation was applied (Krug, 2007). Regarding the Initial lineup, the calculation was only performed with the With perpetrator group (due to the low frequency of correct rejection of the lineup for the Without perpetrator group) and no significant associations were found (rpb = 0.240, *p* = 0.140). In the Final lineup no significant relationships were found, both for the With perpetrator group (rpb = 0.003, *p* = 0.987), and for the Without perpetrator group (rpb = 0.073, *p* = 0.685). In addition, the CAC curves were performed to comprehensively evaluate the confidence-precision relationship. Confidence was divided into low confidence (0–50%), medium confidence (60–80%) and high confidence (90–100%). To calculate the value of the correct proportion corresponding to each confidence level, the following formula was used: # Correct identifications/# Correct identifications + # Incorrect identifications (Mickes, 2015). In the Initial lineup for the target selection, only the With perpetrator group was analyzed, since the other group had no target in the lineup. Correct proportions of 0.11 (*N* = 4), 0.23 (*N* = 8), and 0.05 (*N* = 2) were obtained for low, medium, and high confidence, respectively (Figure 3A). In the Initial lineup for the foil selection, the With perpetrator group presented an incorrect proportion of 0.08 (*N* = 3), 0.38 (*N* = 13), and 0.11 (*N* = 4) in low, medium and high confidence, respectively. The Without perpetrator group presented at low confidence an incorrect proportion of 0.71 (*N* = 7), at medium confidence 0.78 (*N* = 23), and in high confidence an incorrect proportion of 0.66 (*N* = 1) was found (Figure 3B). In the Final lineup for the target selection, the With perpetrator group presented correct proportions of 0.07 (*N* = 3), 0.28 (*N* = 11), and 0.07 (*N* = 3) in low, medium, and high confidence, respectively. The Without perpetrator group presented correct proportions of 0.12 (*N* = 4), 0.09 (*N* = 3), and 0 (*N* = 0) in low, medium, and high confidence, respectively (Figure 3C). Finally, in the Final lineup for the foil selection, the With perpetrator group presented incorrect proportions of 0.17 (*N* = 7), 0.28 (*N* = 11), and 0.10 (*N* = 4) in low, medium and high confidence, respectively. The Without perpetrator group presented incorrect proportions of 0.25 (*N* = 8), 0.46 (*N* = 15), and 0.06 (*N* = 2) in low, medium, and high confidence, respectively (Figure 3D).

### Symptomatology Scales and Lineup Recognition

We divided the symptomatology scales into high and low scores (depression score: high ≥ 9.17, low < 9.17; anxiety score: high ≥ 35.14, low < 35.14; and sleep quality score: high ≤ 6.78, low > 6.78). No significant differences were found between the With and Without perpetrator groups in terms of levels of depression [χ2(1) = 2.36, *p* = 0.12, *V* = 0.18], anxiety at day 1 [χ2(1) = 0.14, *p* = 0.91, *V* = 0.14], anxiety at day 8 [χ2(1) = 0.56, *p* = 0.45, φc = 0.09], and quality of sleep [χ2(1) = 0.21, *p* = 0.65, φc = 0.05]. A summary of the symptomatology scale scores (divided by groups) can be found in Table 2.

**TABLE 2:** Symptomatology scales.

	With perpetrator	Without perpetrator	t (70)	*p*
State anxiety (day 1)	34.927.54	35.399.62	–0.23	0.81
State anxiety (day 2)	32.437.84	32.247.94	0.10	0.91
State anxiety (day 8)	34.878.85	32.758.56	1.02	0.30
Trait anxiety	38.028.76	37.309.31	0.33	0.73
Depression	8.465.89	10.006.56	–1.04	0.29
Sleep quality	6.693.13	6.844.22	–0.21	0.83

*Mean state anxiety (day 1, 2, and 8), trait anxiety, depression, and sleep quality ± SD. A two-tailed *t*-test was used to compare symptom scale scores between the With/Without perpetrator groups.*

No significant associations were found between the performance of the With perpetrator group on the Initial lineup, and levels of anxiety at day 1 [χ2(1) = 0.03, *p* = 0.86, *V* = 0.03], depression [χ2(1) = 0.25, *p* = 0.62, *V* = 0.08] and sleep quality [χ2(1) = 1.1, *p* = 0.30, *V* = 0.17]. None of the high/low subgroups of the three symptom scales achieved target selections above the chance level in the Final lineup.

Once again, due to the low number of correct rejections (2) in the Without perpetrator group, the possibility of making comparisons within this group in the Initial lineup was ruled out.

**FIGURE 4:**
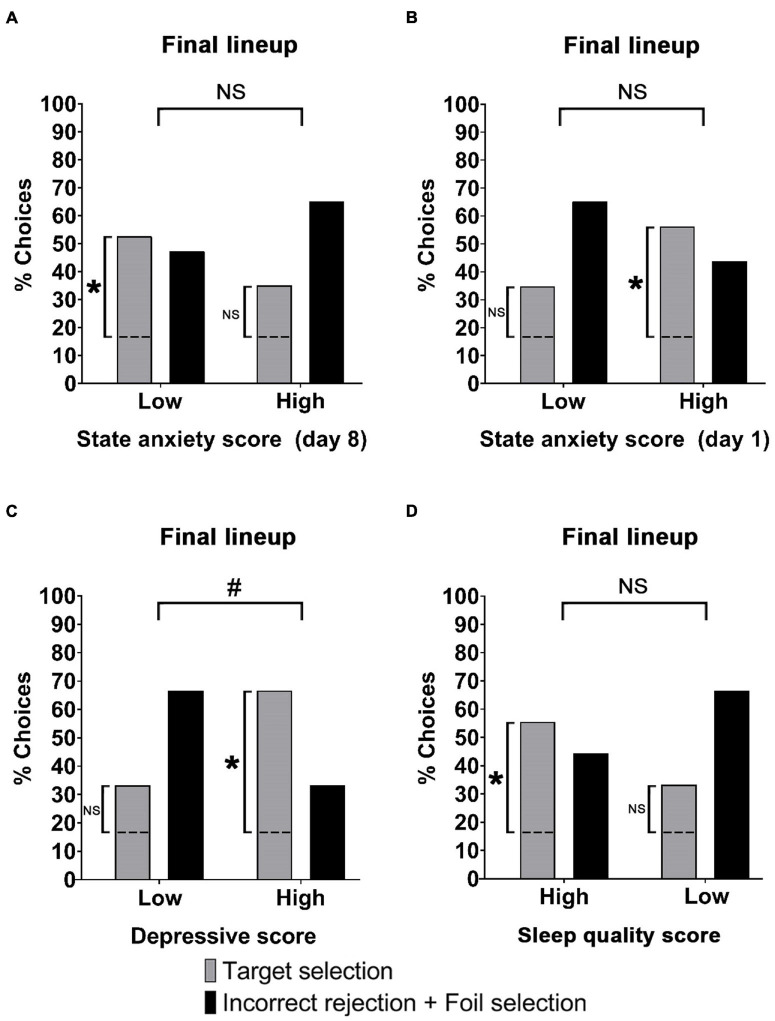
Symptomatology scores and percentage of target selection (correct) and incorrect rejection + foil selection (incorrect) for the With perpetrator group at Final lineup. **(A)** Percentage of correct and incorrect recognitions for high and low anxiety scores on day 8. Low anxiety: correct (*N* = 10) and incorrect (*N* = 9). High anxiety: correct (*N* = 7), incorrect (*N* = 13). **(B)** Percentage of correct and incorrect recognitions for high and low anxiety scores on day 1. Low anxiety: correct (*N* = 9), incorrect (*N* = 7). High anxiety: correct (*N* = 8), incorrect (*N* = 15). **(C)** Percentage of correct and incorrect recognitions for high and low depression scores. Low depressive: correct (*N* = 9), incorrect (*N* = 18). High depressive: correct (*N* = 8), incorrect (4). **(D)** Percentage of correct and incorrect recognitions for high and low sleep quality scores. High sleep quality: correct (*N* = 10), incorrect (*N* = 8). Low sleep quality: correct (*N* = 7), incorrect (*N* = 14). *p < 0.05; NS, *p* > 0.05. The dashed lines stand for the chance level (17% of correct responses). The perpetrator was included in the Final Lineup of both groups.

We further differentiated the identifications made in the Final lineup for the With perpetrator group, dividing the participants in high and low symptomatology scores. We observed no significant differences for the target selection between high and low anxiety score on day 8 [Figure 4A, χ*2*(2) = 1.23, *p* = 0.27, *V* = 0.18], high and low anxiety score on day 1 [Figure 4B, χ*2*(2) = 1.77, *p* = 0.18, *V* = 0.21], high and low depression score [Figure 4C, χ*2*(2) = 3.75, *p* = 0.053, *V* = 0.31], high and low sleep quality score [Figure 4D, χ*2*(2) = 1.95, *p* = 0.16, *V* = 0.22]. However, we find that the target selection for the low anxiety at day 8 were significantly above the chance level [χ*2*(2) = 5.73, *p* = 0.02, φc = 0.39], also for high anxiety at day 1 [χ*2*(2) = 4.8, *p* = 0.03, φc = 0.39], high depression [χ*2*(2) = 6.2, *p* = 0.13, φc = 0.51] and high sleep quality condition [χ*2*(2) = 5.9, *p* = 0.15, φc = 0.41]. No other subgroup achieved target selections significantly above the chance level (1.11 < (χ*2*(2) < 2.13, all *ps* > 0.29).

The same analysis was applied to the Without perpetrator group. We observed no significant differences for the target selection between high and low anxiety score on day 8 [χ2(1) = 0.001, *p* = 0.98, *V* = 0,01], high and low anxiety score on day 1 [Figure 4B, χ2(1) = 0.79, *p* = 0.38, *V* = 0.16], high and low depression score [Figure 4C, χ2(2) = 0.11, *p* = 0.74, φc = 0.06], high and low sleep quality score [Figure 4D, χ2(2) = 1.87, *p* = 0.17, φc = 0.24]. None of the high/low subgroups of the three symptom scales achieved target selections above the chance level in the Final lineup [0 < (χ*2*(2) < 0.85, all *ps* > 0.36].

### Symptomatology Scales and Episodic Memory Recall

**FIGURE 5:**
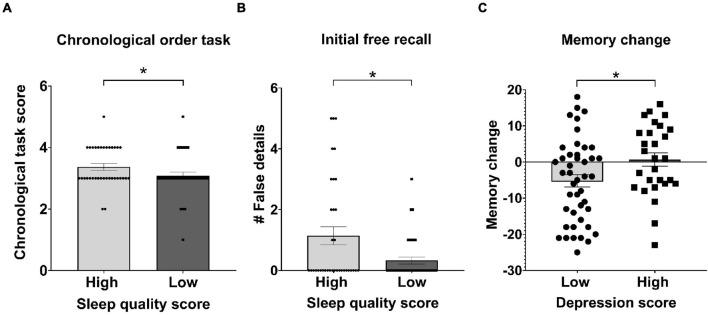
Symptomatology scores and recall. **(A)** Mean of chronological order task score ± SEM, for high and low sleep score. **(B)** Mean number of incorrect details ± SEM on the Initial free recall, for high and low sleep quality. **(C)** Mean of memory change between initial recall and final recall, for high and low depressive score ± SEM. *p < 0.05, ^#^ number of.

We found that those participants with high sleep quality, perform better in the chronological order task than those with low sleep quality (Figure 5A, Mdn = 3, Mdn = 3, respectively. Mann-Whitney *U*-test, *U* = 501.50, *p* = 0.03, RB = 0.23).

In addition, we observed that those participants with high sleep quality, provide a greater number of incorrect details during the Initial recall than those with low sleep quality (Figure 5B, Mdn = 0, Mdn = 0, respectively. One-tailed Mann-Whitney *U*-test, U = 513.50, *p* = 0.03, RB = 0.21). We further found that those subjects who exhibited a high degree of depressive symptoms, reported a smaller drop in the number of details, between the Initial recall and the Final recall with respect to those who showed a low depressive level (Figure 5C, *M* = −0.68, *SD* = 9.77, *M* = 5.23, *SD* = 11.47, respectively. Two-tailed *t*-test (70) = 2.25, *p* = 0.02, *d* = 0.26). No other significant differences were found for the recall variables grouped by the levels of the symptom scales (−1.31 < *t* (70) < 1.29, all *ps* > 0.08).

## Discussion

This study was a first step toward understanding how lockdown by COVID-19 pandemic context may influence eyewitness identifications and episodic memory formation. We first found that participants in the With perpetrator group who exhibited high anxiety on the first day, selected the target in the Final lineup above the chance level. On the contrary, those who showed a low degree of anxiety on day 8, selected the target in the Final lineup above the chance level. These results go in line with the model proposed by Schwabe et al. (2012), pointing out that stress has a differential effect on each memory phase, it facilitates memory acquisition and consolidation but impairs memory recall. However, these results were not observed for the Without perpetrator group which showed no significant difference to the chance level in the selection of the target in the final lineup independent of the level of anxiety. Thus, the level of anxiety seems to moderate encoding and recall only when the target is present in the initial lineup.

Regarding the recall variables, we observed no significant differences between the anxiety level for neither the free recall nor for the Chronological order task. The differences shown by our data in the anxiety modulation between target selection in the lineup and the episodic memory could be due to the influence of uncontrolled variables such as cognitive overload and test expectancy which could be impacting in a different way the different types of recall (Hall et al., 1976; Flindall et al., 2016).

We further observed that those participants within the With perpetrator group who showed a high degree of depression selected the target above the chance level, but participants with low depression did not. A similar result was obtained for the recall variables, where those participants with a high degree of depressive symptoms had a lower decay in memory change between the Initial recall and the Final recall, than those with low depression. Taken together, these results could be explained as the product of a biased processing in favor of negative content (Watkins et al., 1996). Thus, the participants with high depression score would tend to remember the video of a perpetrator entering a conference better than those with low score, given their tendency to strengthen their own negative vision of the world.

Regarding the temporal order, it has been shown that patients with depressive disorder showed an impairment in the temporal order of their episodic memories (Habermas et al., 2008). However, we found no significant differences between high and low scores of depression for the Chronological order task. Although, this discrepancy could be due to differences in the methodology, since our participants exhibit different degrees of depressive symptoms but none of them reached a pathological level.

In addition, we found that those participants within the With perpetrator group who had high quality of sleep selected the target significantly above change in the Final lineup but this result was not found for the participants in the low condition. This is supported by several studies showing that sleep improves memory acquisition and consolidation (Rasch and Born, 2013). However, we did not find any differences for the Without perpetrator group. It is important to highlight that there are only a few studies analyzing the role of sleep on eyewitness lineup identifications (Stepan et al., 2017; Morgan et al., 2019) and they even showed contradictory results. On one hand, Stepan et al. (2017) found that participants that slept between the training and testing sessions rejected the lineup when the perpetrator was absent significantly more than if they stayed awake. They found no significant difference for the condition where the perpetrator was present in the lineup independently of the sleep/wake condition. On the other hand, in a similar procedure, Morgan et al. (2019) did not observe differences between groups of participants that either slept after the training or remained awake. Thus, the differences between our and their studies could be mainly explained by the methodology used. In those studies, a short and controlled period of sleep deprivation is used, while our work is based on prolonged periods of low sleep quality, which arises spontaneously as a consequence of environmental conditions of the COVID-19 pandemic.

Regarding the recall variables, we observed that participants with high sleep quality recalled more incorrect details on day 1 than participants with low quality. We would expect that a low quality of sleep will be related to a decrease in source monitoring inducing more false memories (Fenn et al., 2009). However, it is important to consider that the more information is remembered the greater the probability to form false memories (Otgaar et al., 2019). Although we did not observe a significant increase in correct details on day 1 for the high-quality sleep condition, the mean was higher. Thus, we suggest that this could be affecting false memory formation on day 1.

In addition, we found that those subjects with high sleep quality obtained better results in the Chronological order task. This highlights the widely accepted fact that adequate sleep is conducive to learning (Rasch and Born, 2013). Both, synaptic homeostasis, and active memory consolidation, may explain the better performance of those individuals who sleep adequately (it is likely that both factors act in combination). In this way, an adequate sleep regimen favors not only the acquisition of new information, but also its correct storage and its persistence in time. Furthermore, it has been observed that sleeping after learning emotional stories favors the consolidation of temporal order (Groch et al., 2011).

The results suggest that simply being exposed to an innocent suspect in an intervening lineup, whether that innocent suspect is identified by the witness or not, increases the probability of misidentifying the innocent suspect and decreases the probability of correctly identifying the true perpetrator in a subsequent test lineup.

It has been largely demonstrated that multiple lineups would increase the chance to identify an innocent as a suspect and decrease the probability of correctly identifying the true perpetrator (Hinz and Pezdek, 2001). Here we replicated those findings showing that in the Final lineup, the Without perpetrator group had significantly fewer target selections than the With perpetrator group. This is not surprising, considering that both groups tended to the same extent to repeat their choices in both lineups, but the Without perpetrator almost invariably performed foil selections in the Initial lineup. These results can be explained as a product of the “compromise effect,” since most of the subjects in the Without perpetrator group failed during the Initial lineup (choosing a foil, rather than rejecting the lineup), and 61% of them chose the same foil in the Final lineup (even in front of the real perpetrator), maybe for a compromise with their previous choice. Another possible explanation is the “transference effect.” In our double lineup, a case similar to those observed in Mugshot studies could occur, where the participants remember more vividly the face selected in the Initial lineup (whether it is the perpetrator or not), and then they will tend to repeat their choice during the Final lineup, based on a memory of doubtful origin (not clear if they remember the face of the original event, or the Initial lineup). It is also possible that, in the case of the Without perpetrator group, some features of the foils present during the Initial lineup, which were like those of the perpetrator, generated a prediction error, allowing memory labilization, and causing an updating during reconsolidation, incorporating erroneous information from the faces present in the lineup into the original memory.

When considering the low overall performance of both groups, the possibility of explaining the results because of low encoding level, derived from contextual conditions, should also be considered. It has been extensively documented how an individual’s state at the time of learning can affect their ability to acquire new information, both positively and negatively (Tyng et al., 2017). In this regard, the current context of lockdown due to the COVID-19 pandemic can be considered mentally and physiologically demanding. If, because of this disturbance, the participants of this study arrive at the Initial lineup with weakly encoded information, a larger number of new details would be incorporated, as if it were a second round of learning (in case the original encoding was minimal, we would really be facing a new learning). However, the Without perpetrator group would only have foils available to encode, and this would explain the difference in the performances observed in the Final lineup.

Concerning the confidence-accuracy relationship, the curve did not seem to follow a clear trend (Sauerland and Sporer, 2007). Although previous studies have observed that lack of sleep can negatively affect the strength of the relationship between confidence and accuracy (Blagrove and Akehurst, 2000) our results are not sufficient evidence to conclude that the relationship between precision and confidence may be affected by the set of negative changes in people’s mental health, like changes in the quality of sleep, increased anxiety, and depression.

Among the limitations of our study, it stands out that the size of the sample could have prevented a more detailed and reliable analysis that would take advantage of more subdivisions of the variables. The approach in real context, offered us a more ecological model, but a less controlled environment. As a result, the intervention of variables not contemplated should not be completely ruled out when considering the results. Additionally, unlike our experimental situation, in a real-life episode, where an individual experiences a violent crime, the sleep disturbances are not likely to appear until after the event, so they would not have an impact on the encoding. Finally, not having pre-lockdown measures in our specific population forces us to speculate based on the trend observed in other populations, and although there are strong reasons to think that anxiety, depression, and the quality of sleep were modified during this period, it is not possible to prove it undoubtedly.

The phenomenon of false memories is complex and multi-determined. It is possible that this work has been able to reflect a portion of that complexity, by showing how different contextual and individual variables interact dynamically to end up in a complex result. From this point, it is essential to move toward a more careful handling of each of the multiple factors mentioned. In particular, regarding identification performance, the variation of the exposure times to the initial stimulus, as well as the manipulation of the degree of similarity between the faces that makes up the lineup, would be a promising horizon toward which to advance in future experiments.

## Data Availability Statement

The raw data supporting the conclusions of this article is available in https://zenodo.org/record/5548028#.YVsjLJrMLIV.

## Ethics Statement

The studies involving human participants were reviewed and approved by Ethics Committee of Institute of Translational Medicine Research Alberto C. Taquini Buenos Aires University. The patients/participants provided their written informed consent to participate in this study.

## Author Contributions

FU, CL, MB, and CF made substantial contributions to the conception and design of the work. FU ran the experiments. FU and PF-K performed the statistical analyses. FU, CL and CF contributed by drafting the work. FU, CL, MB, PF-K, and CF contributed to revising it critically. All authors contributed to the article and approved the submitted version.

## Conflict of Interest

The authors declare that the research was conducted in the absence of any commercial or financial relationships that could be construed as a potential conflict of interest.

## Publisher’s Note

All claims expressed in this article are solely those of the authors and do not necessarily represent those of their affiliated organizations, or those of the publisher, the editors and the reviewers. Any product that may be evaluated in this article, or claim that may be made by its manufacturer, is not guaranteed or endorsed by the publisher.

## Footnotes

1
https://innocenceproject.org/
